# Research progress on Th17 and T regulatory cells and their cytokines in regulating atherosclerosis

**DOI:** 10.3389/fcvm.2022.929078

**Published:** 2022-09-21

**Authors:** Qiong Wang, Yurong Wang, Danyan Xu

**Affiliations:** ^1^Department of Cardiovascular Medicine, The Second Xiangya Hospital, Central South University, Changsha, China; ^2^Department of Internal Cardiovascular Medicine, Second Xiangya Hospital, Central South University, Changsha, China

**Keywords:** CD4^+^ T cells, IL-17, Tregs, Th17/Treg balance, atherosclerosis

## Abstract

**Background:**

Coronary heart disease due to atherosclerosis is the leading cause of death worldwide. Atherosclerosis is considered a chronic inflammatory state in the arterial wall that promotes disease progression and outcome, and immune cells play an important role in the inflammatory process.

**Purpose:**

We review the mechanisms of CD4^+^ T subsets, i.e., helper T17 (Th17) cells and regulatory T cells (Tregs), in regulating atherosclerosis, focusing on the role of interleukin (IL)-17, IL-10, and other cytokines in this disease and the factors influencing the effects of these cytokines.

**Results:**

IL-17 secreted by Th17 cells can promote atherosclerosis, but few studies have reported that IL-17 can also stabilize atherosclerotic plaques. Tregs play a protective role in atherosclerosis, and Th17/Treg imbalance also plays an important role in atherosclerosis.

**Conclusion:**

The immune response is important in regulating atherosclerosis, and studying the mechanism of action of each immune cell on atherosclerosis presents directions for the treatment of atherosclerosis. Nevertheless, the current studies are insufficient for elucidating the mechanism of action, and further in-depth studies are needed to provide a theoretical basis for clinical drug development.

Cardiovascular disease due to atherosclerosis has become the leading cause of death worldwide (1). The formation of atherosclerosis is a complex process. low-density lipoprotein (LDL) facilitates lipid accumulation in the arterial wall, endothelial dysfunction allows lipoprotein particles penetrating into the subendothelial layer of the arterial wall, where plaque formation occurs, and local inflammatory response and misbalanced functioning of tissue macrophages contribute to the plaque growth and the formation of lipid core (2–4). Although lipid deposition has previously been considered a marker of atherosclerosis, an increasing number of studies have reported that immune inflammation also plays a crucial role in atherosclerosis occurrence, development, and clinical manifestations (5). The immune inflammatory response is caused by the activation of innate and adaptive immunity (6, 7) and T cell infiltration is present at all stages of atherosclerosis. CD4^+^ T cells are activated by antigens and differentiate into helper T1 (Th1), Th2, Th17, and regulatory T cells (Tregs) (8). The main cytokine produced by Th17 cells is IL-17, It also produces cytokines such as IL-17A, IL-17F, IL-21 and IL-22 (9). And Tregs mainly secrete IL-10 and other cytokines such as IL-35 and TGF-β (10). These CD4^+^ T cell subpopulations can secrete a variety of cytokines to promote or suppress the development of inflammation, thereby regulating atherosclerosis progression. Each of these numerous CD4^+^ T cell subpopulations is involved in the development of immune inflammation. There is growing evidence that Th17 cells and Tregs are highly involved in atherogenesis and the progression of atherosclerosis (11) (Figure 1).

**Figure 1:**
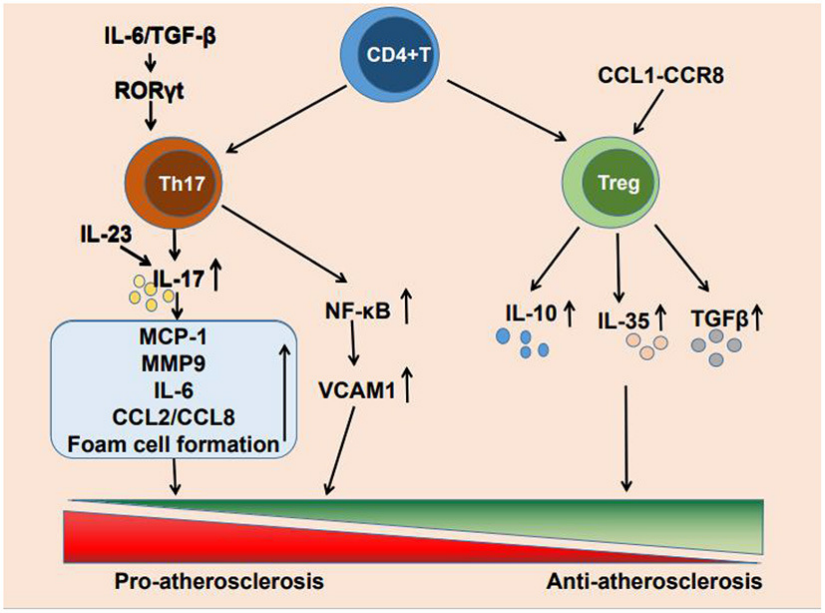
Mechanism of Th17 cells and Treg cells regulating atherosclerosis.

Here, we reviewed the mechanisms of Th17 cell- and Treg-mediated atherosclerosis in existing CD4^+^ T cell subpopulations, thereby providing targets for atherosclerosis treatment and a feasible direction for research on the inhibition of atherosclerosis progression through immunomodulation.

## Th17 cells

Th17 cells are characterized by the expression of interleukin (IL)-17A, IL-17F, IL-21, and IL-22. IL-17 is mainly secreted by Th17 cells and is considered their signature cytokine. Nevertheless, IL-17 can also be produced by various types of hematopoietic cells in addition to Th17 cells and by CD8^+^ T cells, invariant natural killer T cells (iNKT), and other immune cells (12). As a major cytokine that is secreted by Th17 cells, IL-17 plays an important role in regulating the formation of atherosclerosis. The interaction between its pathway and cytokines is complex. Further exploration of its mechanism would provide a new direction for atherosclerosis treatment.

### IL-17 promotes atherosclerosis development

There have been many studies on the role of IL-17 in atherosclerosis development and progression. Wang et al. (13) and Gao et al. (14) reported that ApoE-/- mice exhibited Th17 cells in atherosclerotic plaques and higher percentages of Th17 cells than wild-type (WT) mice, suggesting that Th17 cells are associated with atherosclerosis. Eid et al. (15) detected IL-17 in the serum of patients with coronary atherosclerosis and reported large Large amount of IL-17 production by Th17 cells infiltrating the arteries, suggesting an association of IL-17 with atherosclerosis. Gao et al. (14) injected IL-17 (2 mg/mouse) weekly into ApoE^−/−^ mice. After 5 weeks, the IL-17 injection greatly exacerbated aortic root plaque formation, confirming that IL-17A exacerbated atherosclerosis development *in vivo*. Erbel et al. (16) reported that when co-cultured with atheromatous plaques, IL-17A induced upregulation of the mRNA of proinflammatory mediators, including the chemokine monocyte chemoattractant protein-1 (MCP-1) and the plaque-disrupting matrix metalloprotease 9 (MMP9). It also induced the expression of a proinflammatory transcriptome in macrophages, leading to the upregulation of proinflammatory mediators, including cytokines such as IL-1A and IL-6 or chemokines such as C-C motif chemokine ligand (CCL) 2 and CCL8, thereby promoting atherosclerotic plaque formation. Similarly, Shiotsugu et al. (17) stimulated human umbilical vein endothelial cells with IL-17A and reported that IL-17A induced adhesion molecule expression and promoted monocyte adhesion to vascular endothelial cells. Moreover, IL-17A stimulated oxidized low-density lipoprotein (oxLDL)-induced foam cell formation by upregulating LOX-1 in activated macrophages, thereby participating in atherosclerosis pathogenesis. In addition, IL-17 induced the release of chemokines such as CXCL1, CXCL2, and CXCL8 from endothelial cells and vascular smooth muscle cells (SMCs) (18), and the increase of these chemokines attracted other inflammatory cells to the inflammation site, exacerbating atheromatous plaque inflammation. Similar to a recent study, Zhang et al. (19) reported recently that IL-17A could bind to IL-17RA on the endothelial cell membrane and promote endothelial cell senescence, which is one of the main causes of structural changes and blood vessel dysfunction and the basis of atherosclerosis (20), by activating the nuclear factor kappa B (NF-κB)–p53–Rb signaling pathway.

In contrast, Erbel et al. (21) reported in 2009 that IL-17 antibody neutralization of IL-17A reduced the expression of vascular cell adhesion molecules (VCAM-1), immune cell infiltration, and the secretion of proinflammatory cytokines (IL-6 and tumor necrosis factor [TNF]-α) and chemokines (CCL5), significantly reducing the area of atherosclerotic lesions in ApoE^−/−^ mice (IL-17A monoclonal antibody [mAb]-treated group: 1586 ± 723 μm^2^; control group: 2716 ± 1187 μm^2^), maximum stenosis, and lesion vulnerability, suggesting a proatherosclerotic effect of IL-17. Similarly, Erbel et al. (16) reported that inhibiting IL-17A in ApoE^−/−^ mice slowed the progression of advanced atherosclerotic lesions by reducing the necrotic core of atherosclerosis and that IL-17A mAb treatment slowed the progression of advanced atherosclerotic lesions by increasing fibrous cap thickness, collagen content, and connective tissue growth factor (CTGF) mRNA expression to exert a plaque-stabilizing effect. Recently, Wang et al. (13) injected anti-IL-17 mAb intraperitoneally into ApoE^−/−^ mice for 4 weeks and observed reduced atherosclerotic plaque formation and decreased lesion size, collagen content, and necrotic core in the IL-17-neutralized ApoE^−/−^ mice. These studies suggest that IL-17 acts as a proinflammatory cytokine to promote atherosclerosis development.

### IL-17 mediates atherosclerosis-related mechanisms

#### IL-17 induces VCAM-1 expression in vascular SMCs to promote atherosclerosis

Vascular SMCs play a central role in atherosclerosis. VCAM-1 is expressed in endothelial cells and the intimal SMCs of atherosclerotic lesions and is required for leukocyte recruitment and vascular inflammation (22). VCAM-1^−/−^ mice did not develop atherosclerosis, suggesting that VCAM-1 is required for atherogenesis (23). IL-17 mediates VCAM-1 expression in SMCs through multiple signaling pathways, thereby promoting atherosclerosis development. Zhang et al. (24) demonstrated that IL-17 was dependent on NF-κB to induce VCAM-1 expression in SMCs and that knockdown of p65 with small interfering RNA reversed IL-17-induced VCAM-1 expression, suggesting that NF-κB is indispensable in such expression in SMCs. TGF-β activated kinase 1 (TAK1) is considered an important IL-17-mediated signaling molecule (25), but Zhang et al. reported that knockdown of TAK1 did not reduce IL-17-induced VCAM-1 expression in SMCs, nor did it decrease NF-κB activation, suggesting that IL-17-activated NF-κB is independent of the TAK1 pathway. Similarly, they reported that IL-17-induced NF-κB activation was also not dependent on the Akt1 pathway. Furthermore, they noted that IL-17 promoted VCAM-1 expression in SMCs partly through the ERK1/2–MAPK signaling pathway. Therefore, further studies on the above mechanisms and therefore targeting the IL-17 signaling pathway will provide a new means of intervention for treating vascular diseases.

#### Multiple cytokines regulate IL-17 expression

IL-17A expression is regulated by a variety of factors, including cytokines and growth factors. These factors further regulate atherogenesis by regulating IL-17 expression through their downstream signaling pathways or in concert with other factors.

##### IL-6

The IL-6 receptor is a heterodimer composed of IL-6R and gp130. IL-6 binding to its receptor activates the downstream signal transducer and activator of transcription (STAT) 1 and STAT3 (26). IL-6 exerts direct proatherogenic effects (27, 28) but also exerts proinflammatory effects by inducing Th17 cell differentiation. Retinoid-related orphan receptor γ (RORγ) is a member of the nuclear receptor (NR) superfamily in the human genome, the thymus-specific RORγt plays vital roles in promoting T cell differentiation into the Th17 subtype and regulating IL-17A gene transcription in Th17 cells (29). It was found that IL-6 could acts synergistically with transforming growth factor beta (TGF-β) to induce RORγt expression, thereby promoting Th17 cell differentiation (30). Induction of RORγt under Th17 cell-polarizing conditions is dependent upon STAT3, which perceives and transduces signals from IL-6, IL-23 and other cytokines (29) and IL-6 can upregulate IL-21 expression through the STAT3 pathway, which in turn increases IL-23 receptor and RORγt expression, and the combination of RORγt and STAT3 further promotes IL-17 expression (31), which also inhibits forkhead transcription factor p3 (FOXP3) expression (32), thereby promoting the development of atherosclerosis. Blocking IL-6R led to a significant decrease in Th17 cells and an increase in Tregs (33), further demonstrating the important role of IL-6 in the induction of Th17 cell differentiation.

##### IL-23

IL-23 is a member of the IL-12 cytokine family and is a heterodimer composed of the p19 (specific to IL-23) and p40 (shared with IL-12) subunits. IL-23 is thought to be a major driver of the pathogenic human Th17 cellular response (34). The IL-23–IL-17 cytokine axis drives a variety of chronic inflammatory diseases, including arthritis, psoriasis, and inflammatory bowel disease (35). Abbas et al. (36) reported that IL-23 and IL-23R expression was increased in carotid atherosclerotic plaques and that IL-23 induced a significant increase in the release of IL-17 from the cells of patients with carotid atherosclerosis and accelerated their inflammatory state, suggesting that IL-23 may promote the development of atherosclerosis through the IL-23–IL-17 axis. The Th22 subset is a novel CD4+T-cell subset and the most important source of IL-22 in mice (37). In the middle and late stages of inflammation, IL-22 is pre-dominantly released by Th22 cells to exert immune effects (38). In addition, the study found that IL-23 can induce IL-22 production by CD4^+^ T cells, which may be a downstream signal for IL-23 to act (39). Shi et al. (40) reported that in ApoE^−/−^ mice, plaque size was observed after treatment with recombinant (r)IL-22 and IL-22 mAb, which revealed that Th22 cell-derived IL-22 activated IL-6/STAT3, increased dendritic cell-induced Th17-cell proliferation, stimulated SMC dedifferentiation to a synthetic phenotype, and ultimately promoted the development of atherosclerosis. This further suggests the importance of the IL-23–IL-17 axis in pro-atherosclerosis.

In contrast, it has been suggested that the IL-23–IL-17 axis may play a neutral role in atherosclerosis. The onset of inflammation in psoriasis and atherosclerosis shares the same cytokine pathway (41). The dysfunction in psoriasis includes systemic inflammation involving cytokines, such as IL-17, TNF-α and IFN-γ (42), as well as inflammatory transcripts such as CXCL10, IL-1β and VCAM-1.Psoriasis increases vascular stiffness and atherosclerosis tthrough the IL-17 pathway (43). Animal studies have demonstrated that the transfer of *Ldlr*^−/−^ IL-23R^−/−^ CD4^+^ T cells to *Ldlr*^−/−^ *Rag*1^−/−^ recipient mice resulted in reduced the Th17 levels and IL-17^+^IFN-γ^+^ Th17 cells, suggesting that IL-23 mediates IL-17 production. However, studies showed IL-23-dependent pathogenic Th17 effector cell differentiation exerted no significant effect on atherosclerosis in *Ldlr*^−/−^ mice (44). Furthermore, a 5-year follow-up study of patients with severe psoriasis revealed the occurrence of cardiovascular events after IL-12–IL-23 blockade treatment and that IL-12–IL-23 blockade exerted no effect on cardiovascular events (45). Similarly, Marovt et al. (46) reported the significant efficacy of biologics targeting the IL-23–IL-17 axis in the treatment of psoriasis. However, the effect on vascular structure and function was neutral, suggesting that the IL-23–IL-17 axis may exert no effect on atherosclerosis development.

The above differences in the effects of the IL-23–IL-17 axis on atherosclerosis have not been elucidated and may lie in the models used in studies on the IL-23–IL-17 axis, the different periods in which atherosclerosis occurs, and the impact of other comorbid diseases in patients with atherosclerosis on the studies; or even the different types of IL-23 knockdown in the animal models. In addition, cytokines act on inflammation via complex pathways, and despite IL-23 knockout, whether IL-17 plays a role in atherosclerosis through other unknown pathways has not been ruled out. In conclusion, the existence of these differences suggests that there remain numerous unknown parts of the pathways of cytokine action, and further research to identify these pathways is expected to provide targets for atherosclerosis treatment.

##### Other cytokines

An isoform of the proinflammatory cytokine IL-1 (47), IL-1β is involved in atherogenesis. The Canakinumab Anti-inflammatory Thrombosis Outcomes Study (CANTOS) demonstrated significant reductions in recurrent cardiovascular events after selective neutralization of IL-1β with a monoclonal antibody (canakinumab) in patients who were at risk of residual inflammation (5). Inhibition of IL-1β reduced the sizes of formed atherosclerotic plaques and increased plasma IL-10 levels (48), thereby slowing the onset of atherosclerosis. In addition, Engelbertsen et al. (49) reported that knockdown of IL-1R1, the IL-1β receptor, reduced IL-17A production by stimulated splenocytes and plasma IL-17A levels and attenuated other proinflammatory cytokines secreted by Th17 cells, suggesting that IL-1R1 signaling affects atherosclerosis by promoting Th17 immune response development. In addition, receptor interacting protein 2 (RIP2) and tripartite motif containing (Trim) 21 are associated with Th17 cell differentiation. In *Rip*2^−/−^ mice, T cells preferentially polarized toward pathogenic Th17 cells under pathogenic conditions, resulting in increased IL-17A production, which directly led to the significant enhancement in atherosclerosis (50). MMP expression was reduced in *Trim21*-deficient mice and human plaques (51), and *Trim21* deficiency in hematopoietic cell compartments led to plaque enlargement through increased T cell-mediated IL-17 responses, along with significant increases in plaque collagen content and thicker fibrous cap (52). These studies confirm the proinflammatory effects of multiple factors in inducing Th17 cell differentiation and increasing IL-17 production, but no studies have elucidated their mechanisms of action due to their intricacy. Therefore, further investigation of their mechanisms is of great importance for preventing and treating atherosclerosis.

### IL-17 plays a protective role in atherosclerosis

Although a large number of studies have suggested the proatherogenic effects of IL-17, others have suggested that IL-17 inhibits the development of atherosclerosis. Suppressor of cytokine signaling (SOCS) protein is considered a key physiological regulator of innate and adaptive immunity and SOCS3 plays a protective role in atherosclerosis by inhibiting the STAT3 signaling pathway and suppressing proinflammatory responses (53). Taleb et al. (54) reported that neutralizing IL-17 in T cell-specific SOCS3-deficient mice completely eliminated the atherosclerotic protective effect of T cell-specific SOCS3 deletion and led to significant atheromatous plaque lesion formation. Furthermore, the administration of rIL-17 to *Ldlr*^−/−^ mice significantly reduced the development of atherosclerotic lesions and reduced IL-1-induced endothelial VCAM-1 expression *in vitro*, suggesting that IL-17 inhibits the development of atherosclerosis. Subsequently, Danzaki et al. (55) found in ApoE^−/−^, IL-17A^−/−^ mice that IL-17A deficiency led to atherosclerotic lesions and the formation of unstable atherosclerotic plaques, whereas atherosclerotic plaque formation was reduced in ApoE^−/−^ mice and ApoE^−/−^, IL-17A^−/−^ mice when treated with IL-17A. The authors also suggested that the lack of atheroprotective effects of IL-17A may have been due to increased interferon (IFN)-γ production and decreased IL-5 production in the splenocytes. Although such studies on the anti-inflammatory effects of IL-17 are scarce, its role cannot be ignored, and the elucidation of its complex targets of action yields directions for the immune response to modulate atherosclerosis.

As a cytokine, IL-17 involves numerous pathways and has multiple interactions or influences. Therefore, studies on specific models or different stages of atherosclerosis and different IL-17 concentrations are expected to clarify its mechanism of action in atherosclerosis and provide a feasible and sustainable target for treating such diseases.

## Tregs play a protective role in atherosclerosis

A growing body of evidence emphasizes the role of T cells as important drivers and regulators of atherogenesis (56). Tregs are a CD4^+^ T cell subpopulation that suppress exacerbated inflammatory responses to enhance immune tolerance and homeostasis (57). Tregs can be subdivided into two major categories based on their developmental origin: thymic Tregs and peripherally induced Tregs. T cell differentiation to Tregs is driven by FOXP3 (58), which is essential for Tregs to maintain their immunosuppressive functions. Using an ApoE^−/−^ mouse model, AitOufella et al. (59) demonstrated that CD4^+^CD25^+^ Treg deficiency was associated with a significant increase in atherosclerotic lesion size, demonstrating for the first time that endogenous CD4^+^CD25^+^ Tregs play a protective role in atherogenesis. In contrast, Gao et al. (60) performed selective transfer of CD4^+^FOXP3(GFP)^+^ Tregs into the aorta of ApoE^−/−^ mice and observed that the Treg transmigration reduced the aortic facial atheroma plaque area by 54.3% (*P* < 0.001) and the aortic root plaque area by 34.0%, (*P* < 0.001) and significantly reduced macrophage infiltration (65.2%, *P* < 0.001), suggesting the important role of Tregs in atherosclerosis development.

Tregs maintain immune homeostasis and tolerance through the release of immunosuppressive cytokines (e.g., IL-10 and TGFβ), cell contact-dependent mechanisms, and the promotion of tissue repair (61). Further studies have revealed that Tregs could directly exert their anti-inflammatory effects on monocytes and macrophages. Coculturing monocytes and macrophages with Tregs significantly inhibited the production of proinflammatory cytokines such as TNF-α, IL-6, and IL-1β, while the production of the anti-inflammatory cytokines IL-1RA and IL-10 was enhanced (62), suggesting that Tregs can inhibit proinflammatory cytokine production by acting on monocytes and macrophages. In addition, co-culture with Tregs rendered monocytes less capable of increasing the T cells that secreted harmful IL-17 by decreasing monocyte CD86 expression (63). When Tregs were transferred to ApoE^−/−^ mice, there was a significant decrease in the relative level of macrophage and lipid content in plaques and significant increases in SMCs and collagen, thereby reducing the risk of plaque rupture (64). The mechanism by which Tregs affect various cells associated with atherogenesis is not well defined and exhaustive, and there are still many gaps to be filled.

### Antiatherosclerotic effects of IL-10

IL-10 is an immunomodulatory cytokine mainly produced by Treg, as well as B cells, macrophages and other immune cells (65). The main cytokine secreted by Tregs, IL-10 plays an important role in regulating atherosclerosis.

IL-10 significantly affects the inflammatory response in atherosclerosis. Mallat et al. (66) studied IL-10^−/−^ and IL-10^+/+^ C57BL/6J mice and found that compared to the IL-10^+/+^ mice, the IL-10^−/−^ mice demonstrated a significant 3-fold increase in aortic sinus atherosclerotic lesions and lesions expressing higher levels of IFN-γ, increased activated T cell infiltration, and decreased collagen levels, which increased atherosclerosis while decreasing atheromatous plaque stability, suggesting an important atheroprotective role of IL-10. Subsequently, a study of ApoE^−/−^ mice reported a significant increase in the proinflammatory Th1-cell response and lesion size in IL-10 deficiency, along with an increase in MMP and tissue factor activity in lesions (67), suggesting that IL-10 reduced atherogenesis. Similarly, Fourman et al. (68) measured IL-10 in serum samples with enzyme-linked immunosorbent assay and assessed coronary atherosclerosis using computed tomography angiography in an observational study of human immunodeficiency virus (HIV) patients and uninfected controls, and found that reduced IL-10 was associated with increased coronary plaque incidence and increased carotid intima-media thickness, suggesting a protective effect of IL-10 against atherosclerosis in HIV. Conversely, Liu et al. (69) used adeno-associated virus type 2 (AAV) vector transduction of IL-10 in *Ldlr*^−/−^ mice to upregulate IL-10 gene and protein expression and observed reduced subintimal lipid accumulation and lower CD68 and reactive oxygen species levels, suggesting that systemic AAV vector transduction of the IL-10 gene could suppress inflammation and oxidative stress, thereby inhibiting atherosclerosis. Recently, Kim et al. (70) successfully reversed proinflammatory cytokine production by immune cells in lesions and reduced atherosclerotic plaque progression by targeting IL-10 delivery via nanocarriers to atheromatous plaque sites in ApoE^−/−^ mice fed a high-cholesterol diet. The above studies bifurcated the protective role of IL-10 in atherosclerosis and explored how IL-10 could be more effectively transported to the target site to exert its anti-inflammatory effects. Although the study was successful, the experimental subjects were animals, and further research is needed to translate the results to atherosclerosis treatment in humans.

### IL-35 regulates tregs to play a protective role in atherosclerosis

IL-35 is a cytokine secreted by Tregs and consists of the IL-12p35 and EBV-inducible gene 3 (EBI3) subunits. IL-35 is a reactive cytokine (71) induced by proinflammatory stimuli and is present in the early atherosclerosis development. IL-35 inhibits cardiovascular inflammation effectively through its important role in suppressing endothelial cell activation (72). Tao et al. (73) reported that exogenous human and mouse rIL-35 treatment reduced atherosclerosis in ApoE^−/−^ mice, suggesting that IL-35 has atheroprotective effects. In addition, IL-35 is also involved in regulating Tregs. In an IL-35 treatment model, IL-35 increased splenic Tregs and inhibited atherosclerosis in ApoE^−/−^ mice (73). Moreover, IL-35 promoted C-C motif chemokine receptor 5 (CCR5) expression in the Tregs of ApoE^−/−^ mice (74), increased CCR5 enhance the immunosuppressive function of splenic Tregs in three ways, which including CCR5-mediated Treg migration (possibly from the spleen, which is a large Treg reservoir, to the aorta), inhibit ATK-mTOR signaling in the Tregs, and promote the immunosuppressive function of TIGIT and PD-1 in the Tregs (75). These studies suggested the importance of IL-35 in regulating Tregs to exert atheroprotective effects.

### Other cytokines that regulate Treg function

The CCR8 ligand CCL1 is a cytokine expressed by activated T lymphocytes (76). In the cardiovascular system, CCL1 is expressed in endothelial cells, macrophages, and the extracellular regions of human atherosclerotic plaques (77). CCL1 stimulates vascular SMC migration (78) and activates endothelial cells in response to arterial wall injury (79). CCR8 is mainly expressed in Tregs and plays a key role in immunosuppression by activating Tregs through interactions with its ligand CCL1 (80). CCL1 knockdown exacerbated atherosclerosis in fat-fed ApoE-KO mice, and high-fat-fed ApoE/CCL1-DKO (double knockout) mice demonstrated a reduced percentage of Tregs in the aorta and spleen compared to ApoE-KO control mice, and significantly reduced IL-10 in the splenocytes and plasma, while the proliferative activity of CD4^+^ T cells isolated from the spleen and lymph nodes was not affected. Furthermore, treatment of adipose-fed Ldlr*-*KO mice with CCR8-blocking antibodies reduced Treg levels in the aorta and enhanced aortic atherosclerosis (81). These studies suggested that CCL1–CCR8 axis inactivation decreases Treg recruitment, leading to increased atherosclerosis. In addition, Tregs can also produce TGF-β, which inhibits effector T-cell differentiation; suppresses T- and B-cell proliferation; and inhibits macrophage, dendritic cell, and NK-cell activity to exert a stabilizing effect on atherosclerosis (10).

Studies showed that prolonged exposure to inflammatory cues can promote Treg functional plasticity or affect Tregs stability (82), leading to instability when Tregs lose Foxp3 expression and the loss impairs the suppressive capacity (functionality) of Treg cells. The animal study by Wolf et al. (83). Found that bulk FoxP3+ Tregs from 8-week-old Apoe^−/−^ transferred into 24-week-old Apoe^−/−^ mice mostly lost FoxP3 and started expressing RORγT and T-bet after 6 weeks. And in adoptive transfer experiments, converting ApoB+ Tregs failed to protect from atherosclerosis, suggesting that the instability of Treg cells deprives them of the ability to suppress inflammation. In contrast, the plasticity of Treg cells is beneficial, IFNγ+T-bet+CXCR3+Th1-like Tregs, IL4+IL5+IL13+GATA3+Th2-like Tregs and IL17A+RORγt+Th17-like Tregs have been identified currently (84–86). Studies found that in response to IFNγ or IL-27 Tregs acquire Th1 characteristics by expressing T-bet and CXCR3, preferentially accumulate in Th1 inflammatory niches, and render Th1 cells more susceptible to suppression (87), suggesting that the plasticity of Treg cells enhances their inhibitory ability. Although the mechanism remains unclear, the stability and plasticity of it provide a new idea to regulate atherosclerosis by intervening Treg.

Anti-inflammatory cells that regulate atherosclerosis, Tregs secrete a variety of cytokines that play important roles in all stages of atherosclerosis. Treg pathways and mechanisms of action have been increasingly described, further exploration based on existing studies presents the possibility of immunotherapies for atherosclerosis.

## The role of the Th17/Treg balance in atherosclerosis

Tregs expressing FOXP3 exert anti-inflammatory effects through contact-dependent inhibition or the release of anti-inflammatory cytokines (IL-10 and TGF-β) (88). Proinflammatory Th17 cells expressing RORγt play an important role in the development of autoimmune and allergic reactions via the production of IL-17 and IL-6 (89). Th17-related cytokine (IL-17 and IL-6) and transcription factor (RORγt) levels were significantly higher and Treg numbers, Treg-related cytokine (TGF-β1) and transcription factor (Foxp3) levels were significantly lower in ApoE-/- mice than in age-matched C57BL/6J mice, suggesting that Th17/Treg balance is important (90). Furthermore, Zhu et al. (91) reported significantly higher expression of the Th17 transcription factor RORγt and IL-17 levels in the serum of patients with systemic lupus erythematosus (SLE) combined with atherosclerosis as compared with that in SLE-only patients and control groups, and significantly lower Treg numbers and the expression of the transcription factor Foxp3, further suggesting that Th17/Treg imbalance may play a role in atherosclerosis formation and development. Multiple signals, factors, epigenetic modifications, metabolic pathways, and microbiota can regulate the balance between Tregs and Th17 cells (92).

Multiple factors regulate Th17/Treg homeostasis. CD69 is a C-type lectin, the earliest activation cell surface receptor on leukocytes, expressed by small subsets of T and B cells in peripheral lymphoid tissues (93). CD69+T lymphocytes are detected in cell infiltrates of various chronic inflammatory diseases (94). The lymphocyte activation antigen CD69 regulated Th17 cell and Treg differentiation, where CD69-deficient mice exhibited enhanced Th17 cell differentiation and defective Treg functions (95) and resulting in an increase in atherosclerotic plaque area (96). oxLDL was previously considered to be highly inflammatory and immunogenic (97), whereas recent studies have reported that its binding to CD69 on human T cells induces the upregulation of NR4A receptor expression, which downregulates the percentage of IL-17^+^ cells produced by Th17 cell polarizing stimulation, promotes Treg differentiation, and exerts a protective effect against inflammatory responses (96). Huang et al. (98) reported that IL-12p35 deficiency decreased IL-35 levels and inhibited Treg production and function in ApoE^−/−^ mice, while IL-12p35 deficiency was followed by increased Th17-cell levels and IL-17 levels, thereby exacerbating the Th17/Treg imbalance and promoting atherosclerosis. In contrast, treatment of ApoE^−/−^ mice with human rIL-35 increased circulating and local Treg levels, inhibited the Th17 immune response, and reduced plaque size, suggesting that IL-35 alters the Th17/Treg balance by upregulating Treg immune responses, thereby attenuating atherosclerosis (99). In addition, studies have shown that indoleamine 2,3-dioxygenase (IDO) may play an important role in regulating Th17/Treg homeostasis. Tryptophan (Trp) is an essential amino acid for T-cell differentiation and its metabolite kynurenine (Kyn) inhibits Th17-cell differentiation and induces Treg proliferation. IDO is the rate-limiting enzyme for Trp degradation via the Kyn pathway, which controls Th17 and Tregs transformation by regulating Trp metabolism (100). Yang et al. (101) recently reported reduced IDO activity in P. gingivalis-infected atherosclerotic patients: the activity was negatively correlated with Th17-cell percentages and Th17/Treg ratios and positively correlated with Treg percentages; moreover, reduced IDO activity was accompanied by decreased serum IL-10 levels and increased IL-17 levels. This finding further suggests that IDO is important in regulating the Th17/Treg balance in atherosclerosis patients. Several studies have demonstrated that regulating the Th17/Treg balance by decreasing IL-17 and increasing Foxp3+ cells can reduce inflammatory cell infiltration, inhibit chronic inflammation, and stabilize atherosclerotic plaques (102, 103). In addition, remodeling the gut microbiota can prevent the development of atherosclerosis (104), and immune cells and cytokines can synergize with the microbiota and its metabolites to regulate atherosclerosis and plaque regression (105), moreover, altering the gut microbiota can re-establish the Th17/Treg balance (106). Although several studies have demonstrated that multiple cytokines affect the Th17/Treg balance, the specific mechanisms have not been elucidated, and further clarification of the mechanism would provide a new direction for delaying atherosclerosis by regulating Th17/Treg homeostasis.

## Conclusions and prospects

Atherosclerosis is a major cause of mortality worldwide and its pathogenesis includes lipid infiltration, monocyte macrophage infiltration, injury responses, and the inflammatory responses. In recent years, the role of immune-mediated inflammatory responses in the progression of atherosclerosis has received increasing attention and different types of immune cells play anti-inflammatory or proinflammatory roles in atherosclerosis. Understanding the pathways by which these immune cells and their secreted cytokines act would yield therapeutic directions for preventing the onset and progression of atherosclerosis.

## Data availability statement

The authors confirm that the data supporting the findings of this study are available within the article [and/or] its supplementary materials.

## Ethics statement

Ethical review and approval was not required for this study in accordance with the local legislation and institutional requirements.

## Author contributions

QW and YW initiated this article and wrote the manuscript. QW was the first author of this manuscript. DX revised our first draft and provided valuable comments. All authors contributed to the article and approved the submitted version.

## Conflict of interest

The authors declare that the research was conducted in the absence of any commercial or financial relationships that could be construed as a potential conflict of interest.

## Publisher's note

All claims expressed in this article are solely those of the authors and do not necessarily represent those of their affiliated organizations, or those of the publisher, the editors and the reviewers. Any product that may be evaluated in this article, or claim that may be made by its manufacturer, is not guaranteed or endorsed by the publisher.
